# Detection and Determinants of *Leptospira* Infection in Rodents, Cattle, and Humans in Muheza District, Tanzania: A Call for One Health Approach

**DOI:** 10.1002/puh2.70043

**Published:** 2025-03-24

**Authors:** Gamba Gerald Manyama, Gerald Dickson Mlowe, Athumani Msalale Lupindu, Abdul Suleman Katakweba

**Affiliations:** ^1^ Department of Veterinary Medicine and Public Health Sokoine University of Agriculture Morogoro Tanzania; ^2^ African Centre of Excellence for Innovative Rodent Pest Management and Biosensor Technology Development (ACEII, IRPM‐BTD) of the Sokoine University of Agriculture Morogoro Tanzania; ^3^ Department of Animal, Aquaculture and Range Sciences Sokoine University of Agriculture Morogoro Tanzania; ^4^ Institute of Pest Management Sokoine University of Agriculture Morogoro Tanzania

**Keywords:** leptospirosis | Mgunda | microscopic agglutination test | One Health | serovar | zoonosis

## Abstract

Interaction among humans, livestock, and wildlife plays an important role in zoonotic disease transmission. The emergence of *Leptospira* in humans, rodents, and cattle remains relatively understudied. A cross‐sectional study was conducted between February and May 2023 in Muheza to determine evidence of *Leptospira* infection and associated factors in rodents, cattle, and humans. A total of 479 serum samples from rodents (*n* = 201), humans (*n* = 198), and cattle (*n* = 80) were examined by microscopic agglutination test (MAT) to detect antibodies against 6 live *Leptospira* stock culture serovars, including Pomona, Hebdomadis, Canicola, Grippotyphosa, Sokoine, and Lora. Additionally, a questionnaire survey was conducted on 140 respondents to determine factors that are associated with *Leptospira* seropositivity. Descriptive statistics and Chi‐square test were used to analyze the data. The overall *Leptospira* seroprevalence in rodents, cattle, and humans was 6.0% (12/201; 95% CI: 3.12%–10.20%), 12.5% (10/80; 95% CI: 6.16%–21.79%), and 13.1% (26/198; 95% CI: 8.76%–18.65%), respectively, and the most predominant serovars were Grippotyphosa, Sokoine, and Hebdomadis. A significant difference in the seroprevalence was observed in occupation, whereby farmers were more likely to be infected with *Leptospira* than those in other occupations (*χ* ^2 ^= 9.19, df = 3, *p* = 0.027). This study showed co‐agglutination among rodents, cattle, and humans with serovars Hebdomadis, Sokoine, and Grippotyphosa. People aged 36–59 had the highest seropositivity, suggesting they are the most at‐risk group. This study shed light on pathogenic serovars circulating among humans, rodents, and cattle and factors associated with seropositivity. The findings appeal for multisectoral One Health approach for effective control of *Leptospira* infection and other zoonotic diseases.

## Graphical abstract

This study showed co‐agglutination among rodents, cattle, and humans with serovars Hebdomadis, Sokoine, and Grippotyphosa. This study shed light on pathogenic serovars circulating among humans, rodents, and cattle and factors associated with seropositivity. The findings appeal for multisectoral One Health approach for effective control of *Leptospira* infection and other zoonotic diseases.

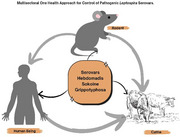

## 1 Introduction

Leptospirosis is a neglected tropical disease (NTD) of public health importance caused by the pathogenic spirochete that belongs to the genus *Leptospira* [1]. *Leptospira* pathogens occur in a variety of forms known as serovars, which are differentiated based on their antigenic characteristics [2]. Globally, approximately 250 *Leptospira* pathogenic serovars have been identified. The disease has been identified as a global public health problem in animals and humans worldwide [3, 4]. It is estimated that severe human cases between 300,000 and 500,000 occur annually, with a mortality rate of up to 30% [5]. Approximately 58,900 human deaths are reported annually [5]. The disease contributes significantly to morbidity and mortality in many sub‐Saharan African countries and disproportionately affects the poor living in rural communities [3]. According to Allan et al. [1], acute human leptospirosis has been recorded in 18 African nations, including Tanzania.

Rodents are among reservoir hosts of *Leptospira* worldwide [6]. Domestic animals, such as cattle, are considered the most important reservoir of *Leptospira*, thus an essential source for human transmission [7]. The common route of transmission to humans is direct or indirect contact with urine or other non‐salivary body fluids of infected domesticated pets and reservoir animals like dogs, pigs, horses, racoons, and wild animals, or contact with contaminated water, soil, or food of infected animals. [8, 9].

In humans, the clinical presentation of the disease is biphasic, with the initial phase being septicemic or acute, and is characterized by a febrile illness lasting almost a week. In the immune phase, antibodies are produced, and leptospires are excreted in the urine [10]. Additionally, the disease can result in miscarriage, stillbirth, maternal death, intrauterine fetal death (IUFD), and congenital infection [11]. In sub‐Saharan countries, about one‐third of pregnant women are estimated to be infected with an NTD agent [12, 13, 14]. Similarly, children under 5 years of age, adults older than65, people with compromised immunity, those with HIV and co‐infections, occupational risks (abattoir workers, veterinarians, farmers, soldiers, and meat vendors), and cancer patients under chemotherapy are at higher risk of *Leptospira* infection [15]. Leptospirosis can be a very severe disease in humans, with a case fatality rate of 5%–15% if left untreated [5].

Tanzania is regarded as one of many countries with favorable environments for survival of *Leptospira* because of its tropical climate [7]. Around 10 serovars have been documented in Tanzania. Of these, six—Sokoine (Icterohaemorrhegia), Serjoe, Hebdomadis, Grippotyphosa, Ballum, and Australis—have been detected across humans, livestock, and wild ungulates in the Katavi and Rukwa regions [16]. Other serovars, including Lora, Canicola, and Pomonanhave, been isolated from cattle and rodents in Morogoro areas [17]. The annual incidence of human leptospirosis is estimated to be 75–102 cases per 100,000 population [18]. About 70% of Tanzanians are engaged in livestock keeping, farming, and fishing activities; thus, they are at high risk of *Leptospira* infection [19]. In recent years, sub‐Saharan African countries, including Tanzania, have experienced periodic outbreaks of human and animal leptospirosis, the most recent being human leptospirosis (Mgunda in Swahili) in Ruangwa, Lindi, whereby three deaths and twenty confirmed cases were reported [7]. Other regions in Tanzania, such as Katavi, have reported a 29.96% prevalence of human leptospirosis [16]. Similarly, 8.8% prevalence in Moshi [20], 13.0% in Kilosa district [21], 12.16% in Mwanza [14], 7.9% in Ngorongoro Conservation Area [22], and 15.1% in Tanga [23]. Additionally, a 13.0%–30.3% prevalence of bovine leptospirosis has been reported in Tanga [24, 25]. Moreover, the prevalence of *Leptospira* antibodies in rodents ranges from 1.8% to 25.8% in Tanzania [26]. The previous research in the study area focused solely on *Leptospira* infection in cattle [23, 24, 27], whereas the present study expands the research spectrum to include humans and rodents. The current study has used six available circulating serovars: Sokoine (Icterohaemorrhegia), Hebdomadis, Grippotyphosa, Canicola, Lora, and Pomona. The result of this study provides crucial information on important components of *Leptospira* transmission cycle and suggests options in design of leptospirosis control plans, including multisectoral, One Health approach.

## 2 Materials and Methods

### 2.1 Description of the Study Area

**FIGURE 1:**
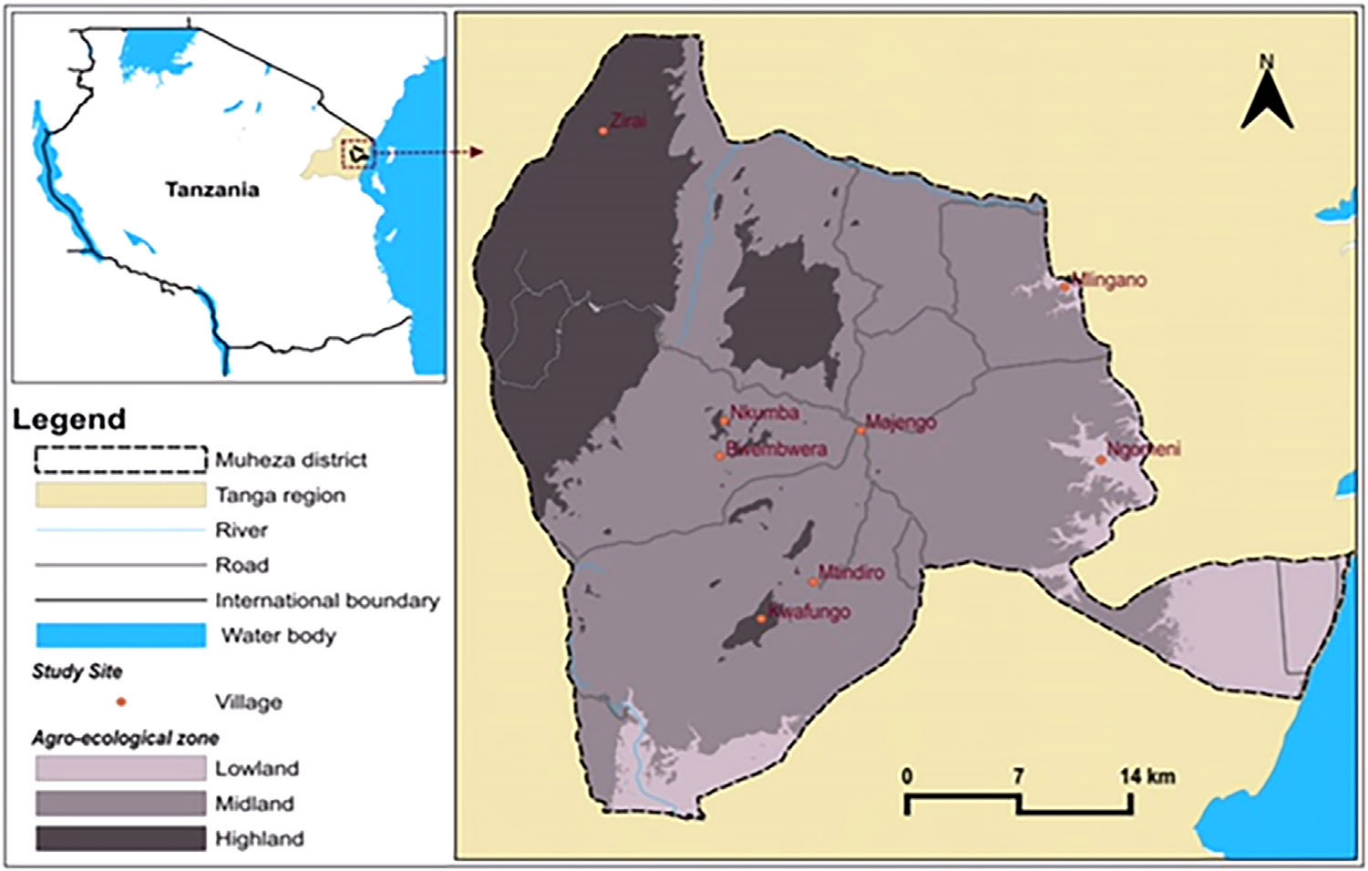
Map of Muheza districts, Tanga region, Tanzania, showing the study sites. *Source:* QGIS. 3.34.1‐Prizren; EPSG: 4326‐WGS 84.

The study was conducted in Muheza district, Tanga (Figure 1). Muheza is one of eight districts of the Tanga region located in north‐eastern Tanzania, at latitudes 40 54′ 18″ S and longitudes 38 0.55′23″ E. The district has a human population of 238,260 and an annual population growth rate of 3.6%, according to the census of 2022 by United Republic of Tanzania [28]. Muheza district is divided into four administrative divisions: Ngomeni, Muheza, Amani, and Bwembwera, and it has a total of 37 wards.

Agriculture remains the mainstay of the district's economy, with over 79% of households involved in agricultural activities. These households depend on rainfed maize, oranges, rice, and vegetables as their main crops, but livestock keeping is another major economic activity. The cattle population in Muheza district is 27,861, of which 9613 are dairy cattle and the rest are indigenous (Muheza District Council).

The study sites were selected on the basis of the agroecological zone, categorized as lowland (75 m and below), midland (75–300 m), and highland (300 m and above). In these agroecological zones, four divisions, including Bwembwera (Kwafungo, Bwembwera, and Nkumba), Muheza (Majengo, Masuguru, Kwemkabala, and Mtindiro), Ngomeni (Mlingano, Mkuzi, Mkanyageni, and Ngomeni), and Amani (Amani, Zirai), were selected (Figure 1). The hospitals were selected on the basis of their proximity to different agroecological zones within the study locality. Eight villages in each division were purposely chosen based on the agroecological zones and the presence of various habitat types (cropland, peridomestic, and indoor). This selection was made to ensure a more comprehensive representation of the study area's overall community of rodents, cattle, and human settlements.

### 2.2 Study Design and Sample Size Estimation

This study employed a cross‐sectional design conducted after the rainy season, from February to May 2023, which involved rodents, cattle, and human sampling. The sample size was estimated using the equation developed by (Cochran WG; [29]). $n=\frac{Z^{2}PQ}{d^{2}}$ [30]. Whereby *n* is the sample size, *Z* = 1.96 (desired confidence level was 95%), *P* is the proportional factor (15.5% for rodents, 15.1% for humans, and 5.6% for cattle) (Schoonman and Swai [25]), *Q* is the 1 − p, and *d* is the desired level of precision (5%). The target was to sample 25 rodents from each of the 8 villages, 10 cattle from each village, and 198 human subjects from Teule Hospital and Ubwari Health Centre. A total of 201 rodents, 198 humans, and 80 cattle were enrolled in this study. One hundred and forty respondents were involved in a face‐to‐face questionnaire interview; among them, 40 were livestock keepers, whereas the rest were patients with the age of 18 and above who visited Teule Hospital and Ubwari Health Centre.

### 2.3 Inclusion and Exclusion Criteria

The study included patients of all ages with febrile illness who visited Teule Hospital and Ubwari Health Centre, live rodents, and cattle older than 1 year. Humans unwilling to consent were excluded from the study.

### 2.4 Rodent Trapping

From March 7 to April 17, 2023, rodent trapping was carried out in houses and around human dwellings, farms proximal to human settlements such as cultivated cropland by using Sherman live traps (7.5 × 9.0 × 23.0 cm^3^) and locally made live traps with wooden box wire mesh window (12 × 15 × 20 cm^3^) [31]. A total of 250 traps (200 Sherman live traps and 50 local traps) were placed per site in 10 lines each with 10 trapping stations, 10 m apart in each station and each line for four consecutive nights. Traps were daily baited by using a mixture of peanut butter and maize brans. In cropland, traps were positioned near holes, irrigation canals, and rat trails. In indoor trapping, traps were placed in feed areas, kitchens, and shelves where food was stored [32]. The traps were set at 1700 h evening and inspected early in the morning at 07:00 h. Captured rodents were collected, anesthetized with diethyl ether preserved with ethanol, and identified to species level using the established taxonomic nomenclature. Rodent morphometric data, including weight, total length, tail length, hind foot length, and ear length, were then recorded [13, 33].

### 2.5 Blood Sampling From Rodents and Cattle

From rodent capture, 1–2 mL of blood was aseptically collected from the heart puncture or plexus by using sterile syringes and needles.

In cattle, which were randomly sampled from different herds from March 17 to 30, 2023, 4 to 10 mL of blood was aseptically collected from the jugular using sterile syringes and needles after proper manual animal restraint.

The samples were immediately transferred into plain vacutainer tubes and allowed to clot for serum separation at room temperature for at least 30 min [34]. The samples were centrifuged for 10 min at 3000 rpm to increase the serum volume. The harvested sera were subsequently transferred into well‐labeled Eppendorf tubes and then stored at −20°C until subjected to microscopic agglutination test (MAT) [33].

### 2.6 Study Humans and Blood Sample Collection

The present study was carried out among individuals with febrile illness who were visiting Teule Hospital and Ubwari Health Center. The participants were asked for consent to take part in the study and that their blood would be screened for leptospirosis. A total of 198 blood samples were collected from consented subjects who visited Teule Hospital and Ubwari Health Centre from May 15 to 26, 2023, for various diagnostic tests, including malaria and typhoid. A 2–4 mL blood sample was aseptically collected from each study subject by medical personnel using sterile syringes [33]. Socio‐demographic information of study participants, including sex, occupation, location, and age, was recorded.

### 2.7 Microscopic Agglutination Test

*Leptospira* stock cultures of the serovars Pomona, Hebdomadis, Canicola, Grippotyphosa, Sokoine, and Lora were purified by sub‐culturing into Ellinghausen–McCullough–Johnson–Harris (EMJH) medium. Pure *Leptospira* cultures were sub‐cultured and incubated for 5–7 days at 30°C before conducting MAT. The purity of the *Leptospira* serovars was checked by dark field microscope for adequate density and the absence of contaminating bacteria. The recommended maximum *Leptospira* density for MAT is 3 × 10^8^ cells/mL on the MacFarland scale [33]. All wells of a microtiter plate were filled with 50 µL phosphate‐buffered saline (PBS), pH 7.2. Except the wells of row 2 that contained 90 µL of PBS. Ten microliters of sera samples were added to the wells of row 2 (dilution was 1:10), followed by serial double dilution with PBS to acquire initial dilutions of 1:10, 1:20, 1:40, 1:80, and 1:160 by pipetting 50 µL from the wells of row 2 to the next rows. At last, the remaining 50 µL were discarded. Afterward, volumes of 50 µL of *Leptospira* antigen were added to all wells of the microtiter plate for initial screening [35]. All plates were incubated for 2 h at 30°C before reading the results. The antigen–serum mixtures were observed under a dark field microscope by taking a drop of antigen PBS mixture to a microscopic slide. Positive samples titer was recorded by detecting 50% *Leptospira* agglutination and living 50% of cells free [36]. Additionally, the sample was considered positive for the infection if it had MAT titers of 1:160 in humans. Similarly, samples with titers of 1:20 and 1:80 were considered exposed to *Leptospira* bacteria. In contrast, samples with MAT titers greater than 1:40 in cattle were regarded as positive. Missed agglutinations were recorded as negative.

### 2.8 Statistical Analysis

Descriptive statistics, such as proportions and percentages for different variables, were computed in Excel. Seroprevalence was determined as a proportion of number of positive samples to the number of samples tested. Chi‐square test was used to assess the association of seropositivity to different variables such as sex, occupation, residence/location, and age category in humans and species in rodents. A *p* value of less than 0.05 was regarded as significant.

### 2.9 Ethical Approval

The research ethical clearance for conducting this study was approved by the Research Ethics Committee at Sokoine University of Agriculture (Ref. No. SUA/ADM/R.1/8/967) issued on December 29, 2022. The permission to conduct research in Muheza was obtained from the Medical Research Coordinating Committee of the National Institute for Medical Research with reference number NIMR/HQ/R.8a/Vol. IX/4269 issued on April 18, 2023. Moreover, before commencing data collection in the study area, authorization was granted from all local authorities, including Muheza district's medical officer (DMO) and village chairmen. Human participants were verbally informed that their blood might be collected and tested for leptospirosis, and verbal consent was obtained from them.

## 3 Results

### 3.1 Rodent Species Captured in the Study Area

A total of 201 rodents were captured from indoor, farmland, and peridomestic habitats. Five different species of rodents, including *Acomys* spp., *Lemniscomys* spp., *Mastomys natalensis*, *Rattus rattus*, and *Tatera* spp., were captured. Out of the rodents sampled, 96 were females. Remarkably, *R. rattus* was the most frequent species captured in the study area. On the contrary, the least relative abundances were found in *Lemniscomys* spp.

### 3.2 Seroprevalence of *Leptospira* Antibodies in Rodent Species

The overall seroprevalence of *Leptospira* antibodies in this study was 6.0% (12/201; 95% CI: 3.12%–10.20%). The highest infection rate was found in *R. rattus* at 4.0% (8/201), followed by *M. natalensis* at 1.5% (3/201), and the lowest was in *Acomys* spp. as shown in Table 1. However, the difference in seropositivity between rodent species was not statistically significant (*p* = 0.3318). Further, the study revealed the variation of seroprevalence of *Leptospira* serovars among rodents to habitat types (*p* < 0.00) and locations (*p *< 0.000) (Table 1).

**TABLE 1:** Rodent species were captured, and their seroprevalence of leptospiral antibodies in various species of rodents in the Tanga region, Tanzania (*n* = 201).

Species	No. of captured and tested	Sex		No of positive	% of positive within species
		F	M		
*Acomys* spp.	7	2	5	1	0.5
*Lemniscomys* spp.	2	0	2	0	0.0
*Mastomys natalensis*	51	19	32	3	1.5
*Rattus rattus*	126	67	59	8	4.0
*Tatera* spp.	15	8	7	0	0.0
Total	201	96	105	12	6.0

### 3.3 Seroprevalence of *Leptospira* Antibodies in Cattle in the Study Area

The seroprevalence of *Leptospira* antibodies in cattle was 12.5% (10/80; 95% CI: 6.16%–21.79%). The variation of seropositivity for sex, grazing system, and location is shown in Table 2.

**TABLE 2:** Seroprevalence of *Leptospira* antibodies in cattle in the study area (*n* = 80).

Parameters	Categories	Number positives	Percentage prevalence
Sex	Cow	3	3.8
	Bull	7	8.8
Grazing pattern	Extensive	7	8.8
	Zero	3	3.8
Location (division)	Amani	1	1.3
	Bwembwera	1	1.3
	Muheza	3	3.8
	Ngomeni	5	6.3

### 3.4 Seroprevalence and Socio‐Demographic Characteristics of Humans

The seroprevalence and socio‐demographic characteristics of humans are shown in Table 3. It was found that out of 198 participants, 48.5% (96/198) were females. The rest were males. The seroprevalence was 13.1% (95% CI: 8.76%–18.65%). A seroprevalence of 7.58% (15/198) recorded among the farmers was significantly high compared to other occupations (*χ* ^2 ^= 9.1894, df = 3, *p* = 0.02688).

**TABLE 3:** Number and percentage of study subjects found seropositive to *Leptospira* bacteria with socio‐demographic information of the study participants (*n* = 198).

Variable	Category	No. and (%) of participants examined	No. and % of participants tested seropositive	Chi‐square (*χ* ^2^)	*p* value
Sex	Female	96 (48.5)	12 (6.06)	0.656	0.418
	Male	102 (51.5)	14 (7.07)		
Occupation	Business	46 (23.2)	10 (5.05)	4.771	0.549
	Employee	26 (13.1)	0 (0.00)		
	Farmers	100 (50.5)	15 (7.58)		
	Students	26 (13.1)	1 (0.50)		
Location	Amani	33 (16.7)	4 (2.02)	2.115	0.549
	Bwembwera	17 (8.6)	3 (1.50)		
	Muheza	60 (30.3)	7 (3.50)		
	Ngomeni	88 (44.4)	12 (6.06)		
Age	18–35	107 (54.0)	10 (5.05)	48.864	0.284
	36–59	80 (40.4)	15 (7.58)		
	≥60	11 (5.6)	1 (0.50)		

### 3.5 Seroprevalence of Different Leptospiral Serovars in Rodents, Humans, and Cattle

Analysis of leptospiral serovars shows that the predominant serovar in humans was Grippotyphosa at 6.06% (12/198), whereas serovar Canicola was not detected (Table 4). In rodents, the predominant serovar was Sokoine at 3.48% (7/201), and serovar Canicola was not detected. Similarly, the predominant serovar in cattle was Grippotyphosa at 6.25% (5/80), whereas serovars Pomona, Canicola, and Lora were not detected (Table 4). Furthermore, the statistical analysis shows that there was no statistically significant difference in the seroprevalence of *Leptospira* serovars among cattle to grazing pattern (*χ* ^2 ^= 1.847, df = 1, *p* = 0.1741), age (*χ* ^2^ = 4.4976, df = 9, *p* = 0.8757), location (*χ* ^2 ^= 5.0286, df = 3, *p* = 0.1697), and sex (*χ* ^2 ^= 1.6162, df = 1, *p* = 0.2036). A higher seropositive proportion was observed in female cattle than in males (Table 2). Similarly, a higher percentage of seropositive cases was observed in extensive grazing areas (*R*) compared to the zero‐grazing pattern (*Z*) (Table 2).

**TABLE 4:** Seroprevalence of different leptospiral serovars in rodents, humans, and cattle in the Tanga region, Tanzania.

Species	Rodents		Humans		Cattle	
Serovars	No of positive	%	No of positive	%	No of positive	%
Pomona	1	0.50	1	0.51	0	0.00
Hebdomadis	2	1.00	7	3.54	4	5.00
Canicola	0	0.00	0	0.00	0	0.00
Grippotyphosa	4	1.99	12	6.06	5	6.25
Sokoine	7	3.48	9	4.55	4	5.00
Lora	1	0.50	1	0.51	0	0.00
Total	15	7.47	30	15.17	13	16.25

### 3.6 Proportions of *Leptospira* Antibody Titers in Cattle, Humans, and Rodents

**FIGURE 2:**
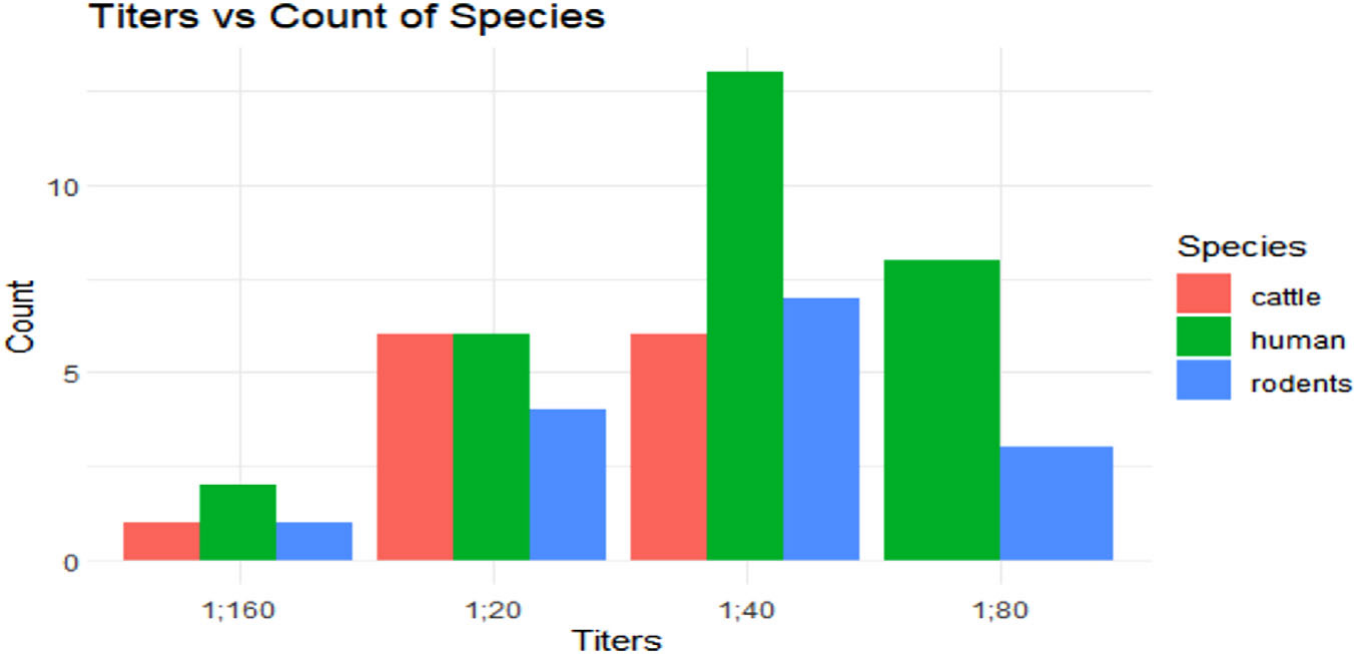
Proportion of *Leptospira* antibody titers in cattle, humans, and rodents in Muheza.

The highest antibody level observed was 1:160. This level was found in two human samples for the Sokoine serovar and one sample each for the Grippotyphosa and Pomona serovars in rodents and cattle. However, the 1:160 level was not found in the Hebdomadis, Canicola, and Lora serovars. The most common antibody level was 1:40, which was most frequently observed in humans. The 1:60, 1:40, and 1:80 were also commonly found in humans, whereas the 1:20 level was most common in both humans and cattle. The 1:80 level was not found in cattle (Figure 2). Serovar Grippotyphosa showed high frequencies for all titers 4.59% (*n* = 22), followed by serovar Sokoine 4.18% (*n* = 20) (Table 4). Serovar Canicola was not detected in the study area.

### 3.7 Multiple Reactions of *Leptospira* Serovars in Rodents, Humans, and Cattle

**FIGURE 3:**
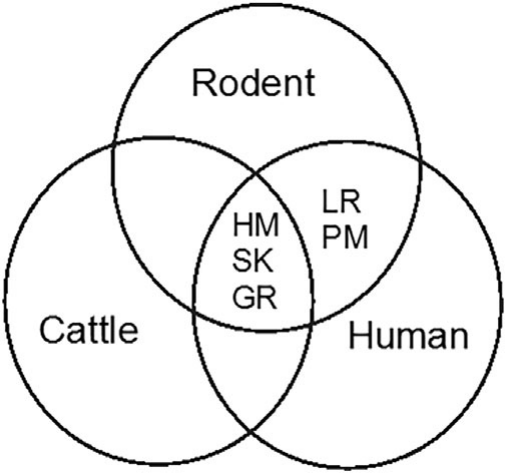
Multiple reactions of *Leptospira* serovars in rodents, humans, and cattle.

Out of 479 rodents, cattle, and humans’ sera that were tested against 6 *Leptospira* serovars, 8 (1.67%) serum samples reacted with more than 1 serovar. In this study, multiple reactions in rodents, cattle, and humans occurred with serovars Hebdomadis, Sokoine, and Grippotyphosa. Furthermore, multiple reactions between rodents and humans occurred with serovars Lora and Pomona only, and none was observed in cattle. No multiple reactions with serovar Canicola were found in any of the three hosts (Figure 3).

### 3.8 Comparison of Seroprevalence of Leptospiral Infection Among Different Variables

Overall, the statistical analysis showed that the variation of seroprevalence of *Leptospira* serovars among rodents, humans, and cattle was significant (*χ* ^2 ^= 6.0046, df = 2, *p* = 0.04967). However, individual species did not show significant differences: rodents (*χ* ^2 ^= 2.3713e − 30, df = 1, *p* = 1), cattle (*χ* ^2^ = 2.7517, df = 1, *p* = 0.09715), and humans (*χ* ^2 ^= 0.001994, df = 1, *p* = 0.9644).

## 4 Discussion

The present study has found that the seroprevalence of *Leptospira* antibodies in rodents is 6.0%. This finding is higher than 1.9% reported earlier [37] and lower than 20.29% reported from Katavi region [16] and 16.9% in Morogoro [38]. On the other hand, the seroprevalence of 12.5% in cattle in the present study is lower than 30.37% reported in Katavi [16] and higher than 6.6% reported in Kigoma and Kagera regions [40]. The seropositivity of 13.1% in humans in the current study is lower than 29.96% from Katavi [16], 30.1% in Endulen, Tanzania [22], and 29.9% in Katavi [39]. On the other hand, the results of the present study are higher than 10.75% from a study in Morogoro [38] and 11.1% reported from Kigoma and Kagera regions [40]. In all these reports, MAT was the method used to determine the seroprevalence. The differences in seropositivity in rodents, cattle, and humans could be due to variation in ecology of the study sites. Moreover, there were some differences in *Leptospira* reference serovars used, which might have led to the differences in the outcome. The current study in Muheza, Tanga, used six references serovars: Sokoine, Lora, Pomona, Hebdomadis, Canicola, and Grippotyphosa. A study in Morogoro [38] also used six serovars: Sokoine, Kenya, Mwogolo, Lora, Canicola, and Grippotyphosa. In a study in Kigoma and Kagera, seven serovars were used, which include Sokoine, Kenya, Sejroe, Grippotyphosa, Bataviae, Lora, and Pomona. In another study in Morogoro, Kilimanjaro, Mbeya, Tanga, Singida, Mwanza, Ruvuma, and Rukwa [37], six *Leptospira* serovars, namely, Sokoine, Harjo, Canicola, Pyogenes, icterohaemorrhagiae and Grippotyphosa, were used. The use of one serovar in one study and not in another could be a possible cause of the differences.

The current study has involved rodents (wildlife), cattle (livestock), and humans. Antibodies against Hebdomadis, Grippotyphosa, and Sokoine serovars were detected in all host species, that is, rodents, cattle, and humans. This study differs from previous studies in study area [23, 27], which investigated seroprevalence of *Leptospira* antibodies in cattle only. As a result, the burden, risk population, and transmission dynamics were difficult to quantify. The current study is similar to studies conducted on rodents, livestock, humans, and wildlife in Katavi–Rukwa ecosystem [16], Bahi, Dodoma [13], and Kilombero, Morogoro [40]. This scenario gives a clue that there is a possibility of sharing of *Leptospira* exposure, something that was previously speculated [13]. Therefore, the findings of the present study call for One Health approach in planning and implementation of *Leptospira* control, whereby personnel from veterinary, medical, wildlife, and environmental sectors and the community have to team up.

The present study has reported multiple agglutinations in 8 sera samples (1.67%) in rodent, cattle, and humans by MAT. MAT is a reference diagnostic tool for *Leptospira* infection. It can discriminate current or active infection from chronic exposure, but it cannot distinguish between co‐infection and cross‐reaction in the case of multiple reactions [41, 42, 43]. In the present study, the serum with highest antibody titer in multiple coagulations was assumed to be the infecting serovar, similar to procedures in other studies [44]. In other words, it was not possible to tell if the multiple coagulations represented multiple infections or an infection and cross‐reaction. To resolve this misnomer, the isolation of *Leptospira* by culture method or molecular typing is recommended.

Detection of *Leptospira* antibodies in *M. natalensis* and *Acomys* spp. in this study underscores the risk to public health. Similar findings have been reported in Kibondo and Kakonko, Kigoma [45]. These rodent species live outside the human settlement and can easily come into contact with livestock or contaminate the environment. As such, they may be a potential source of human leptospirosis [1, 38]. Therefore, there is a need for control of rodents within and around human residence.

The current study found that farmers had the higher *Leptospira* seroprevalence than other occupations, indicating a higher likelihood of being infected with *Leptospira* than other groups. This is especially so, considering the close human contact with animals (rodents and cattle), when sharing common environment; watering points, habitats, and unsecured feeds [46]. The finding of this study is in‐line with other previous studies that have highlighted farmers as being the highest risk groups for leptospirosis in parts of Tanzania and elsewhere [22, 23, 47]. Other researchers have highlighted various groups, including dog keepers [48], livestock keepers [24], fishing communities [33], abattoir workers [49, 50], as well as miners and sewage workers [47], being the other risk groups for contracting leptospirosis Swai, E.S., Schoonman, L., & Daborn, C., [51]. Therefore, there is a need for creation of awareness on this occupational risk to farmers.

The present study has revealed that the predominant serovar stimulating antibody production in humans was Grippotyphosa, followed by Sokoine. This finding is in agreement with reports from studies on predominance of these serovars in humans [13, 14, 23, 24, 47]. Grippotyphosa serovar is naturally habored by cattle [50], whereas rodents are well‐known reservoirs of Sokoine serovar [13]. The detection of antibodies for Grippotyphosa and Sokoine serovars in humans as well as in cattle and rodents suggests the presence of close contact among humans, cattle, and rodents, and possibly sharing of the bacteria. Therefore, in order to reduce this public health threat, rodent control strategies and education to cattle keepers on *Leptospira* infection risk are recommended.

Different levels of antibody titers have been reported in this study. The 1:40 titer was most abundant in humans compared to other titers. This suggests that humans have been exposed to a specific serovar, triggering the production of IgM at high concentration. A lower titer in humans implies a chronic exposure to *Leptospira* that is associated with the presence of IgM [52]. On the other hand, the higher titer of 1:160 was observed in two sera of humans, one rodent, and one cattle serum. This suggests that there was active or acute *Leptospira* infection [38]. The presence of both chronic and acute exposures in the study area implies that *Leptospira* infection risk is a long‐standing threat that calls for intervention.

### 4.1 Limitations of the Study

In Africa, the highest numbers of panel serovars in detecting *Leptospira* antibodies by MAT is 10 serovars [14]. The current study may have underestimated the seroprevalence of *Leptospira* antibodies because only six serovars were included in the panel.

### 4.2 Conclusion and Recommendations

Cattle, rodents, and humans in the Muheza district are infected with five out of the six circulating *Leptospira* serovars, including possible incidences of co‐infection. The findings give a clue on the complexity of a possible *Leptospira* transmission cycle in the study area and other similar areas.

The detection of high *Leptospira* antibodies in rodents, cattle, and humans suggests acute infection. We recommend a multisectoral One Health approach in control of *Leptospira* infection that involves veterinary, medical, wildlife, and environmental personnel as well as the community through rodent control measures, public awareness campaigns, occupational safety protocols for farmers, and other workers who are at great risk of exposure and infection.

## Author Contributions

**Gamba Gerald Manyama**: writing – original draft, conceptualization, methodology, data curation, software, investigation, validation, formal analysis, project administration, funding acquisition, writing – review and editing, visualization, resources. **Gerald Dickson Mlowe**: software, data curation, validation, formal analysis. **Athumani Msalale Lupindu**: conceptualization, methodology, data curation, investigation, validation, supervision, visualization, writing – review and editing, project administration, software. **Abdul Suleman Katakweba**: conceptualization, methodology, investigation, validation, formal analysis, supervision, project administration, resources, funding acquisition, visualization, writing – review and editing.

## Conflicts of Interest

The authors declare no conflicts of interest.

## Data Availability

Raw data will be available upon request.
